# Time to stop using age-based weight estimation

**DOI:** 10.29045/14784726.2021.6.6.1.53

**Published:** 2021-05-01

**Authors:** Giles N. Cattermole, Mike Wells

**Affiliations:** King’s College Hospital NHS Trust; University of the Witwatersrand

We were interested to read Charlton, Capsey and Moat’s (2020) paper on paediatric weight estimation by UK ambulance personnel, and we agree with their conclusion that age-based methods of estimating weight are inaccurate. It appears from their study that of all children aged 1–11, the proportion of estimates within 10% of actual weight (P10) was only 37%, and the proportion of estimates within 20% of actual weight (P20) was only 60%. This is very similar to previous findings of several age-based weight estimation formulae, with typically P10 = 35%, P20 = 65% and P30 = 80% (Cattermole & Manirafasha, 2020; Wells et al., 2017). To put this in clinical context, two thirds of children would be given drugs more than 10% off the correct dose, one third more than 20% and one fifth more than 30%. In contexts (such as the UK) with increasing obesity, it is more likely that these errors will be underestimates of weight. So with morphine, for example, there would be a clinically significant underdosing of a statistically significant proportion of children. This is not acceptable.

Fine-tuning age-based formulae will not help, as has already been well demonstrated; increasing obesity in the community increases not just the mean weight in each age group, but also the range of weights, making age-based rules increasingly imprecise. However, there are alternative weight-estimation tools that are far more accurate. In both those studies cited above, the most precise methods were parental estimate (P10 = 67–80%) and the PAWPER tape (P10 = 62–87%). We disagree with Charlton et al.’s (2020) final conclusion: in pre-hospital or low-resource settings where weight cannot be measured with scales, we would strongly advise against the continued use of age-based estimation methods. Rather than a ‘page for age’, have a ‘page for weight’, and use a more reliable method to estimate that weight. Ask the parent, or use the PAWPER tape.

## Conflict of interest

Mike Wells designed the PAWPER tape, which is referred to in the letter, but it is non-profit and therefore there are no financial conflicts of interest.

## References

[bibr_1] Cattermole G. N. & Manirafasha A. (2020). Accuracy of weight estimation methods in adults, adolescents and children: A prospective study. *Emergency Medicine Journal*. Advance online publication. https://doi.org/10.1136/emermed-2020-209581. 10.1136/emermed-2020-209581 32943398

[bibr_2] Charlton K. Capsey M. & Moat C. (2020). Is weight just a number? The accuracy of UK ambulance paediatric weight guidance – findings from a cross-sectional study. *British Paramedic Journal*, 5(3), 1–7. 10.29045/14784726.2020.12.5.3.1 PMC7783958 33456391

[bibr_3] Wells M. Goldstein L. N. & Bentley A. (2017). The accuracy of emergency weight estimation systems in children: A systematic review and meta-analysis. *International Journal of Emergency Medicine*, 10, 29–72. 28936627 10.1186/s12245-017-0156-5 PMC5608658

